# Identification of potential druggable targets for endometriosis through Mendelian randomization analysis

**DOI:** 10.3389/fendo.2024.1371498

**Published:** 2025-01-22

**Authors:** Peng Chen, Xin Wei, Xiao-Ke Li, Yi-Hang Zhou, Qi-Fang Liu, Ling Ou-Yang

**Affiliations:** ^1^ Department of Obstetrics and Gynecology, Shengjing Hospital of China Medical University, Liaoning, Shenyang, China; ^2^ Department of Obstetrics and Gynecology, Fushun Central Hospital, Liaoning, Fushun, China

**Keywords:** endometriosis, Mendelian randomization, drug target, PPI network, enrichment analysis

## Abstract

**Introduction:**

Endometriosis (EM) is a widely recognized disorder in gynecological endocrinology. Although hormonal therapies are frequently employed for EM, their side effects and outcome limitations underscore the need to explore the genetic basis and potential drug targets for developing innovative therapeutic approaches. This study aimed to identify both cerebrospinal fluid (CSF) and plasma protein markers as promising therapeutic targets for EM.

**Methods:**

We utilized Mendelian randomization (MR) analysis to explore potential disease-causing proteins, utilizing genetic datasets from genome-wide association studies (GWAS) and protein quantitative trait loci (pQTL) analyses. We applied a range of validation techniques, including reverse causality detection, phenotype scanning, Bayesian co-localization (BC) analysis, and external validations to substantiate our findings. Additionally, we conducted a protein-protein interaction (PPI) network as well as functional enrichment analyses to unveil potential associations among target proteins.

**Results:**

MR analysis revealed that a decrease of one standard deviation (SD) in plasma R-Spondin 3 (RSPO3) level had a protective effect on EM (OR = 1.0029; 95% confidence interval (95% CI): 1.0015–1.0043; P = 3.2567e-05; Bonferroni P < 5.63 × 10^−5^). BC analysis showed that RSPO3 shared the same genetic variant with EM (coloc.abf-PPH4 = 0.874). External validation further supported this causal association. Galectin-3 (LGALS3; OR = 0.9906; 95% CI: 0.9835–0.9977; P = 0.0101), carboxypeptidase E (CPE; OR = 1.0147; 95% CI: 1.0009–1.0287; P = 0.0366), and alpha-(1,3)-fucosyltransferase 5 (FUT5; OR = 1.0053; 95% CI: 1.0013–1.0093; P = 0.002) were detected as potential targets for EM in CSF. PPI analysis showed that fibronectin (FN1) had the highest combined score. Furthermore, several EM-linked proteins were involved in the glycan degradation pathway.

**Discussion:**

In conclusion, this comprehensive study offers valuable insights into potential drug targets for EM, with RSPO3 emerging as a promising candidate. Additionally, mechanistic roles of FN1, glycan degradation pathway, LGALS3, CPE, and FUT5 in EM warrant further investigation.

## Highlights

Endometriosis (EM) is a widely recognized disorder in the field of gynecological endocrinology. Hormonal therapies have potential side effects. Our Mendelian randomization analysis suggests that inherent levels of circulating RSPO3 may be causally associated with EM risks. Protein targets identified through our analysis may have promising applications as potential drug targets in EM. CSF galectin-3 may serve as a pain relief target in patients with EM. Further studies are warranted to uncover the precise role of these candidate proteins in EM.

## Introduction

Endometriosis (EM) is a widely recognized disorder in the field of gynecological endocrinology. It is pathologically characterized by the abnormal growth of endometrial tissue in the outer regions of the uterus (1), resulting in chronic severe pelvic pain (2), and pregnancy-associated complications in 5-10% of reproductively active females (3). EM can also lead to infertility in some patients. Types of pelvic pain symptoms in EM-affected women include dysmenorrhea, non-menstrual pelvic pain, dyspareunia, pain at ovulation, dysuria, and dyschezia (4).

EM imposes a significant burden on public health due to its impact on various aspects of women’s lives. EM can negatively affect health-associated quality of life and work productivity, irrespective of the nationality and ethnicity of patients (5). EM increases the risk of other comorbid conditions, especially chronic diseases such as cardiovascular diseases, autoimmune diseases, and cancers (6). The delay in diagnosing EM in the primary healthcare setting further exacerbates the risks of death in these patients (5). Guidelines for early diagnosis and precision treatments can improve the rate of EM-associated death in female patients (6). Often, EM patients suffer from various behavioral disorders, including depression, anxiety, and emotional stress (4), which can significantly compromise their social interactions, sexual lives, and mental health, further contributing to the overall social burden on an individual (4). The economic burden of EM management is also noteworthy and may be comparable to treatment costs of other chronic conditions such as diabetes mellitus, Crohn’s disease, and rheumatoid arthritis (7). The direct costs of EM diagnosis, treatment, and management, combined with a range of indirect costs like lost productivity and reduced work capacity, can contribute to the overall economic and social burden of the patient (8).

Medical treatments for EM typically include a combination of hormonal and surgical interventions. Hormone therapy suppresses the growth and activity of abnormally grown endometrial tissues, while surgery focuses on the removal or excision of endometrial implants and adhesions. Hormonal therapy, as the first-line treatment for EM, can restore the estrogen level, thereby inhibiting the abnormal growth of the endometrium and alleviating associated symptoms. Commonly used hormone therapy medications are oral contraceptives, gonadotropin-releasing hormone (GnRH) agonists, progestins, and aromatase antagonists (9). These medications can help reduce pelvic pain, dysmenorrhea, and other EM symptoms. However, these therapeutics induce certain serious side effects, such as obesity, mood disorders, and irregular menstrual bleeding syndromes. Notably, long-term administration of hormonal medications, such as GnRH agonists, can lead to osteopenia and may not fully alleviate EM symptoms (10–12). Instead, symptoms can recur after discontinuation of hormonal therapeutics.

Current medical treatment options for EM primarily involve hormonal therapies, such as oral contraceptives and progestins (10–13), which often have many side effects; thus, non-hormone medicines are worth noting. Therefore, there is an urgency for developing alternative, possibly non-hormonal treatments, to improve the EM treatment outcomes. For example, alternative and complementary medicines with anti-angiogenic properties may be effective in treating EM (14). Also, modulation of the steroid hormone signaling via DNA hydroxymethylation in EM suggests a potential avenue for non-hormonal treatment strategies (15). However, further investigation is needed to assess the safety and efficacy of these alternative therapeutics.

Using Mendelian randomization (MR), researchers can assess genetic variations in functionally characterized genes to examine the causal relationship between modifiable exposures and their outcomes. MR significantly reduces the impact of confounding factors such as reverse causation in epidemiologic observational studies (16). Recently, MR analysis has been widely utilized for drug target identification and repurposing strategies (17). Advances in high-throughput genomics and proteomics in analyzing cerebrospinal fluid (CSF) and plasma have facilitated the identification of promising therapeutic targets for many diseases (18–21). However, to date, only a limited number of studies have explored the integration of MR with genome-wide association study (GWAS) and protein quantitative trait loci (pQTL) in the context of EM. Although the EM pathogenesis may not be induced by an altered CSF proteomic profile, analysis of the CSF proteomic profile could offer critical clues to the pain symptoms of EM patients. Here, we sought to identify both CSF and plasma protein markers as promising therapeutic targets for EM.

## Materials and methods

### Study design

**Figure 1:**
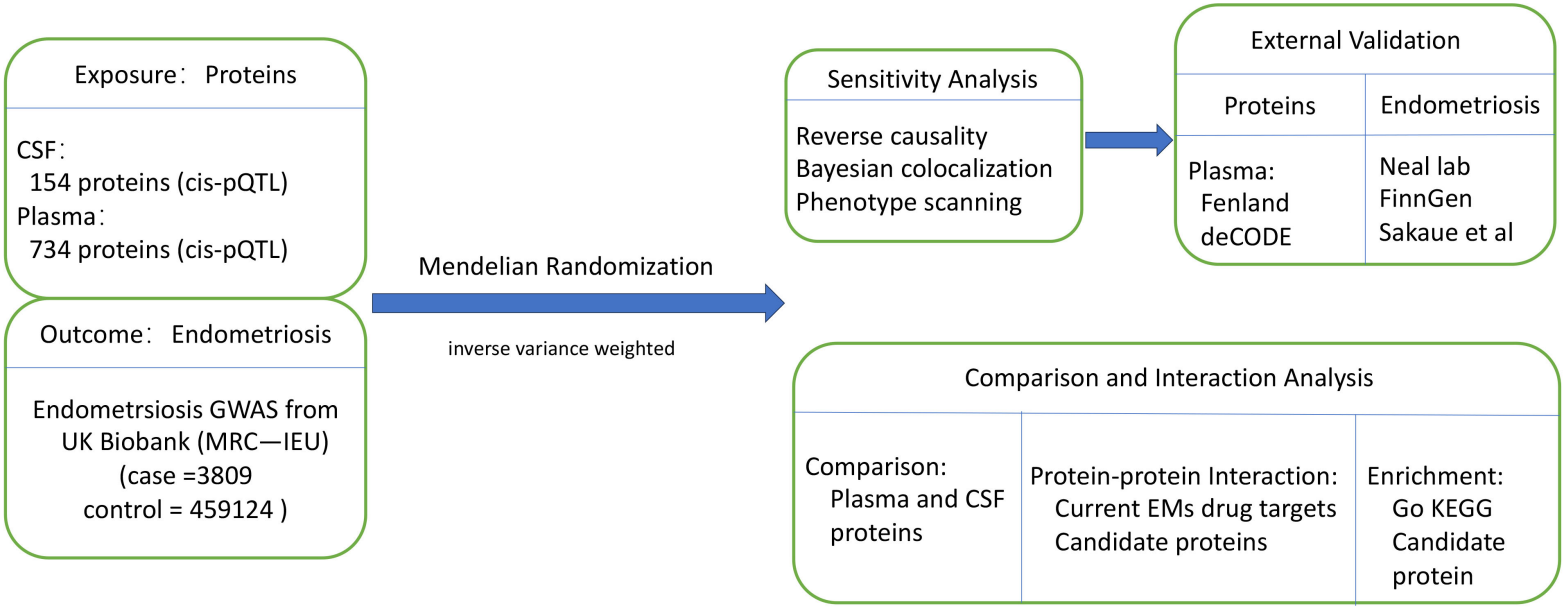
Study design for identification of plasma and CSF proteins causally associated with EM. CSF, cerebrospinal fluid; EM, endometriosis.

The study was based on publicly available data from previous studies. Overall study design is shown in **Figure 1**. First, a two-sample MR analysis was conducted to estimate the causal effects of plasma and CSF proteins on EM, where we derived 154 CSF cis-pQTLs for 154 proteins and 738 plasma cis-pQTLs for 734 proteins from studies by Zheng et al. (22) and Yang et al. (23) and obtained genetic associations of EM from the MRC-IEU with 462,933 of European ancestry in the UK Biobank (24). Only cis-pQTLs were utilized as instruments due to their direct involvement in the transcriptional or translational processes of protein-coding genes. Second, the primary findings were validated by reverse causality (RC) detection, Bayesian co-localization (BC) analysis, and phenotype scanning. Third, using GWAS data and pQTL from other studies (25, 26), we repeated this analysis for external validation to establish our observations. Fourth, we constructed a protein-protein interaction (PPI) network of plasma and CSF proteins as well as between the protein factors and drug targets of EM medications. Lastly, we performed functional enrichment analyses for the identified proteins. The methods and study participant details were outlined below. Our study followed the guidelines of the Strengthening the Reporting of Observational Studies in Epidemiology using Mendelian Randomization (STROBE-MR) (27).

### Data sources and pQTL selection

#### Plasma pQTL and CSF

Plasma and CSF pQTL data were respectively retrieved and obtained from studies by Zheng et al. (22) and Yang et al. (23). These two studies analyzed integrated data from five other GWAS (28–32). Based on the inclusion criteria, pQTLs: (i) with genome-wide significant association (P<5 × 10^−8^); (ii) locating outside the major histocompatibility complex (MHC) genomic locus on chr6; (iii) exhibiting independent association [linkage disequilibrium (LD)-clumping with r^2^< 0.001]; and (iv) with cis-acting functionality, were selected for further analyses. The analysis revealed 154 CSF cis-pQTLs for 154 proteins and 738 plasma cis-pQTLs for 734 proteins (**Supplementary Table S1**).

Furthermore, the plasma pQTL data retrieved from large cohort studies by Ferkingstad et al. (26) and Pietzner et al. (33), were utilized for external validations.

### GWAS summary statistics of EM

We exploited publicly available GWAS summary datasets for non-cancer illness code (self-reported) - endometriosis from MRC-IEU (total n = 462933; case =3809, control = 459124) of European ancestry in the UK Biobank (24). Summary statistics from the FinnGen cohort (R9 release, total n = 77257; case =8288, control = 68969) (34), UK Biobank (total n = 361194; case =1496, control = 359698) (24), and Sakaue et al. (total n = 231771; case =4511, control = 227260) (25) were used for the external validation.

### Statistical analyses

#### MR analysis

We conducted MR analysis of the plasma/CSF proteins as the exposure and EM as the outcome using ‘TwoSampleMR’ (https://github.com/MRCIEU/TwoSampleMR). For a condition where only one pQTL was assigned to a given protein, the Wald ratio was calculated. While for >2 genetic instruments, inverse variance-weighted MR (MR-IVW) and heterogeneity analysis were applied (35). For increased risks of EM, an odds ratio (OR) was determined per 10-fold increase in CSF protein levels or standard deviation (SD) increment in plasma protein levels. The OR for plasma and CSF proteins were defined differently because the data were derived from distinct studies published by Zheng et al. (22) and Yang et al. (23), respectively.

Bonferroni correction was applied in primary analysis to adjust for multiple comparisons, with a threshold P-value < 5.63 × 10^−5^. In a same-variant strategy, the same single nucleotide polymorphism (SNP) found in the primary analysis was used as the genetic instrument (**Supplementary Table S2**); for the significant-variant strategy, genome-wide significant SNPs were used as genetic instruments to validate preliminary observations. P values in the range from 5.63 × 10^−5^ to 0.05 suggested significant associations. Only initially screened protein factors were included in MR analysis for external validation. The threshold value used for the external validation was P < 0.05.

### RC detection

To detect potential RC, genetic instruments for EM were selected in a similar strategy to pQTL selection from GWAS datasets (UK Biobank) for bidirectional MR analysis (**Supplementary Table S3**) (36). Complete summary statistics can be found elsewhere (29). The effect was estimated by MR-Egger, MR-IVW, simple and weighted mode, and weighted median. Steiger filtering ensured the directionality of the protein-EM association (37). A P < 0.05 indicated statistical significance.

### BC analysis

BC assesses the probability of sharing the same causal variant by two traits using the ‘coloc’ package (https://github.com/chr1swallace/coloc). BC determines the posterior probability of hypotheses (PPH) of sharing a single variant between two traits (32). Here, we tested the third PPH (PPH3), stating that both the protein and EM could be associated with regions of different variants, and PPH4, stating that the associated regions could be of shared variants, using the coloc.susie and coloc.and algorithms. We defined a gene as having evidence of co-localization if PPH4>80% in at least one algorithm (35, 38).

### Phenotype scanning

The phenotype scanning was carried out by searching previous GWAS via ‘phenoscanner’ to reveal the association of an identified pQTL with other traits (39). A pleiotropic SNP was considered to possess: (i) a significant genome-wide association (P < 5 × 10^−8^); (ii) a relationship with European descendants in GWAS; and (iii) associations with any known etiological risk factors, including protein, metabolic and clinical traits, of EM. Additionally, the LD r^2^ among pQTLs of prioritized proteins revealed their potential linkages with EM pathogenesis.

### Comparison analysis and PPI network

Considering the blood-brain barrier (BBB), we speculated that there might be minimal correlations between plasma- and CSF-originated pQTLs. Therefore, the correlation between the shared pQTLs was assessed by Spearman correlation, and different P-value thresholds were tested to determine the strength of this correlation.

The PPI network analysis (STRING v12; https://string-db.org/; minimum interaction score = 0.4) was used to detect any associations between EM-linked plasma and CSF proteins. Furthermore, we explored possible interactions between EM-associated genes and drug targets (Drugbank; https://www.drugbank.ca), revealing 23 disease-modifying drugs for EM (40).

### Functional enrichment analyses

Gene Ontology (GO) and Kyoto Encyclopedia of Genes and Genomes (KEGG) pathway enrichment analyses were performed to explore the funcitons and pathways of potential pathogenic proteins related to EM pathology.

### Data availability

GWAS summary statistics for cis-pQTL can be found in original studies (23, 26, 28–32), and that for EM can be retrieved from https://gwas.mrcieu.ac.uk/datasets/. Access to the FinnGen (R9) is available at https://www.finngen.fi/en/access_results.

## Results

### Proteomic analysis of EM-linked proteins

**Figure 2:**
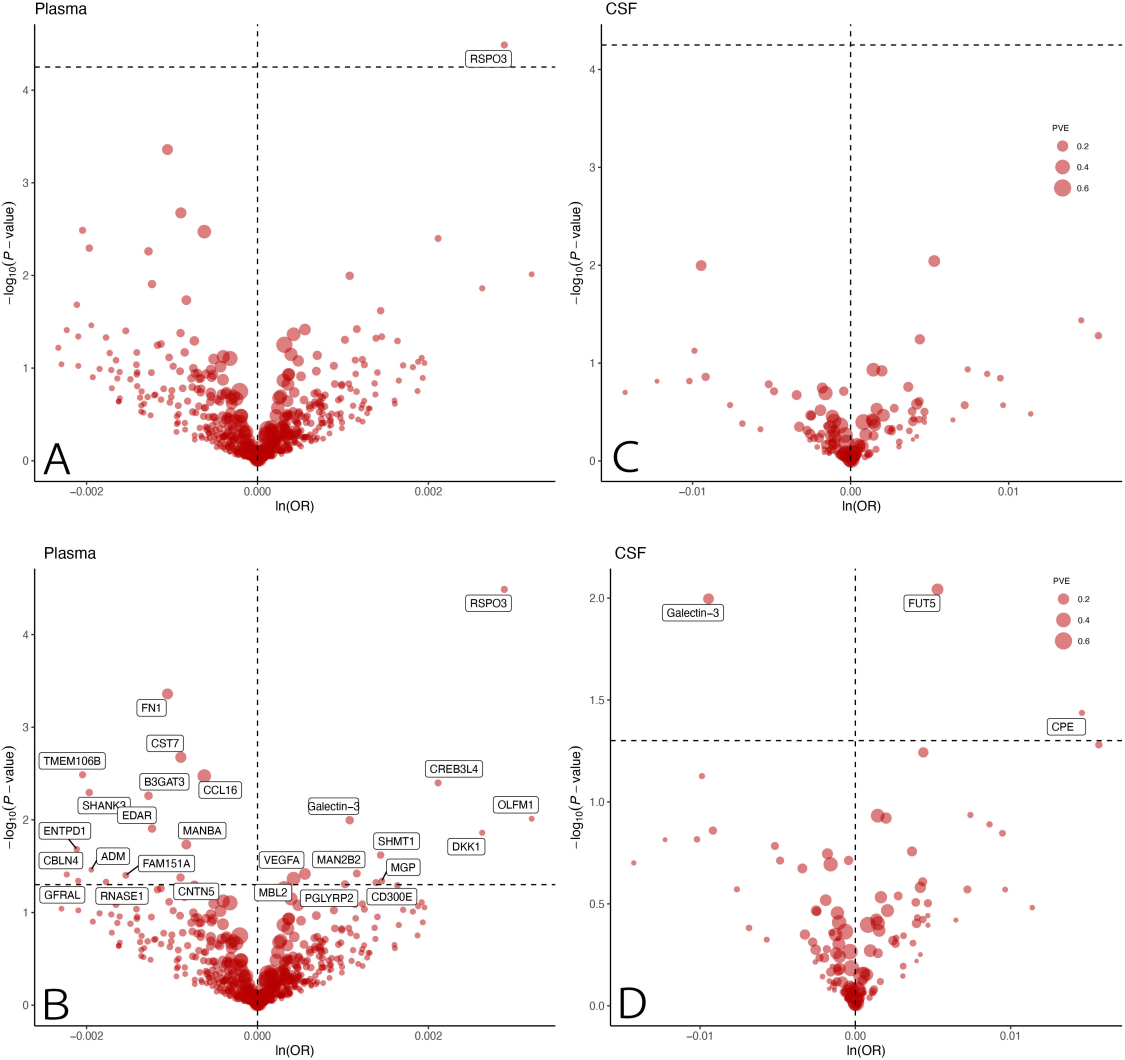
MR results for plasma and CSF proteins and the risk of EM. Volcano plots of the MR results for **(A, B)** 734 plasma and **(C, D)** 154 CSF proteins on the risk of EM. MR analysis with inverse variance weighted method showed the effects of plasma and CSF proteins on the risk of EM, respectively. OR for increased risk of MR were expressed as per SD increase in plasma protein levels and per 10-fold increase in CSF protein levels. **(A, C)** dashed horizontal black line corresponded to P = 5.63 × 10−5 (0.05/888). **(B, D)** dashed horizontal black line corresponded to P=0.05. ln, natural logarithm; PVE, proportion of variance explained; MR, Mendelian randomization; CSF, cerebrospinal fluid; EM, endometriosis; OR, odds ratio; SD, standard deviation.

The MR analysis showed that only one plasma protein R-Spondin 3 (RSPO3) was causally associated with EM (OR = 1.0029; 95% CI: 1.0015–1.0043; P = 3.2567 × 10^−5^) at Bonferroni significance (P < 5.63 × 10^−5^) (**Table 1**; **Figures 2A, C**). MR analysis revealed significantly connected 26 protein-EM pairs in the plasma, and 3 protein-EM pairs in the CSF (P < 0.05) (**Table 1**; **Figures 2B, D**). Among 29 protein-EM pairs, galectin-3 (LGALS3) appeared in both plasma and CSF (OR = 1.0011; 95% CI: 1.0003–1.0019; P = 0.0101, and OR = 0.9906; 95% CI: 0.9835–0.9977; P = 0.0101, respectively); carboxypeptidase E (CPE) and alpha-(1,3)-fucosyltransferase 5 (FUT5) were found in the CSF (OR = 1.0147; 95% CI: 1.0009–1.0287; P = 0.0366, and OR = 1.0053; 95% CI: 1.0013–1.0093; P = 0.002, respectively).

### Sensitivity analysis of EM-associated proteins

**Figure 3:**
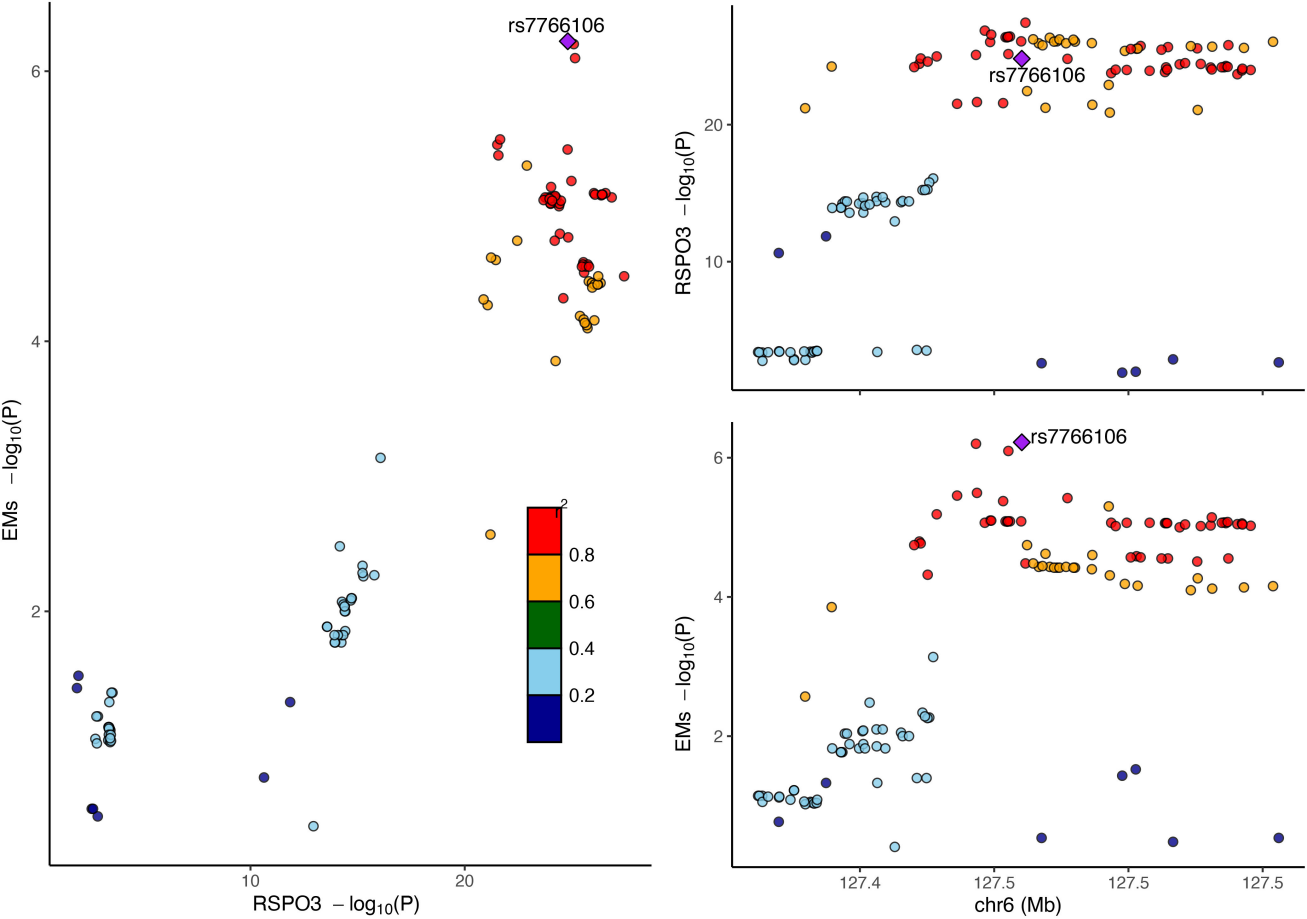
Bayesian colocalization analysis of RSPO3 and EM. Diamond purple points represented the SNP with the minimal sum of P value in RSPO3 GWAS and EM GWAS. EM, endometriosis; GWAS, genome-wide association studies; SNP, single nucleotide polymorphism.

The RC analysis could not identify any causal effects of EM on RSPO3 (IVW, β = 2.4418, P = 0.8382), and Steiger filtering ensured the directionality (**Table 1**). Next, BC analysis indicated that RSPO3 (coloc.abf-PPH4 = 0.874) shared the same variant with EM (**Figure 3**). Furthermore, in phenotype scanning, RSPO3 (rs2489623) expression was found to have associations with waist and hip circumferences, red blood cell (RBC) count, fasting insulin level, triglyceride (TG) level, varicose veins of lower extremities, fracture, heel bone mineral density and chymotrypsinogen B level (**Supplementary Table S4**). Notably, the conditional MR analysis confirmed these associations (**Supplementary Table S4**).

**Table 1:** MR results for plasma and CSF proteins significantly associated with Endometriosis.

Tissue	Protein	UniProt ID	SNP^a^	Effect allele	OR (95% CI)	P value of MR results	PVE	F statistics	Steiger direction	Steiger P value	Author
Plasma	Galectin-3	P17931	rs9323280	A	1.0011 (1.0003, 1.0019)	1.01E-02^*^	7.52%	275.996	TRUE	2.03E-58	Folkersen [32]
Plasma	MGP	P08493	rs7135211	G	1.0015 (1.0000, 1.0029)	4.58E-02^*^	2.76%	194.529	TRUE	9.30E-42	Yao [30]
Plasma	MAN2B2	Q9Y2E5	rs2301790	G	1.0012 (1.0001, 1.0023)	3.79E-02^*^	5.64%	197.178	TRUE	1.34E-42	Sun [29]
Plasma	FN1	P02751	rs1250258	C	0.9989 (0.9984, 0.9995)	4.37E-04^*^	19.78%	245.578	TRUE	3.20E-50	Suhre [28]
Plasma	DKK1	O94907	rs1194673	A	1.0026 (1.0005, 1.0047)	1.38E-02^*^	1.46%	48.8392	TRUE	1.60E-11^*^	Sun [29]
Plasma	MANBA	O00462	rs227370	C	0.9992 (0.9985, 0.9999)	1.85E-02^*^	13.36%	509.157	TRUE	8.31E-105	Sun [29]
Plasma	CCL16	O15467	rs112689088	C	0.9994 (0.9990, 0.9998)	3.36E-03^*^	38.41%	2058.62	TRUE	0.00E+00	Sun [29]
Plasma	CST7	O76096	rs6138458	A	0.9991 (0.9985, 0.9997)	2.11E-03^*^	20.95%	264.174	TRUE	8.57E-54	Suhre [28]
Plasma	CNTN5	O94779	rs1461672	T	0.9991 (0.9982, 1.0000)	4.19E-02^*^	7.40%	79.5288	TRUE	3.47E-18	Suhre [28]
Plasma	RNASE1	P07998	rs17254387	A	0.9982 (0.9965, 1.0000)	4.68E-02^*^	2.11%	71.1026	TRUE	2.33E-16	Sun [29]
Plasma	MBL2	P11226	rs7899547	G	1.0004 (1.0000, 1.0008)	4.31E-02^*^	40.00%	2201.1	TRUE	0.00E+00	Sun [29]
Plasma	VEGFA	P15692	rs6921438	A	1.0006 (1.0000, 1.0011)	3.83E-02^*^	24.43%	1067.01	TRUE	1.01E-208	Sun [29]
Plasma	SHMT1	P34896	rs8067462	C	1.0014 (1.0002, 1.0027)	2.40E-02^*^	4.31%	144	TRUE	1.58E-31	Emilsson [31]
Plasma	ADM	P35318	rs2923091	A	0.9981 (0.9963, 0.9999)	3.45E-02^*^	0.96%	66.5856	TRUE	4.83E-15	Yao [30]
Plasma	ENTPD1	P49961	rs11188501	A	0.9979 (0.9961, 0.9997)	2.07E-02^*^	1.99%	67.1524	TRUE	1.98E-15	Sun [29]
Plasma	CD300E	Q496F6	rs8081669	G	1.0014 (1.0000, 1.0028)	4.76E-02^*^	3.52%	116.64	TRUE	6.10E-26	Emilsson [31]
Plasma	B3GAT3	O94766	rs12794886	C	0.9987 (0.9978, 0.9996)	5.49E-03^*^	7.83%	280.563	TRUE	3.27E-59	Sun [29]
Plasma	GFRAL	Q6UXV0	rs72975088	T	0.9979 (0.9959, 1.0000)	4.56E-02^*^	1.50%	50.2551	TRUE	6.12E-12	Sun [29]
Plasma	CREB3L4	Q8TEY5	rs4845586	G	1.0021 (1.0007, 1.0036)	3.99E-03^*^	3.12%	106.223	TRUE	2.02E-23	Sun [29]
Plasma	FAM151A	Q8WW52	rs11206397	T	0.9985 (0.9970, 0.9999)	3.96E-02^*^	3.03%	103.166	TRUE	4.40E-23	Sun [29]
Plasma	PGLYRP2	Q96PD5	rs55866012	T	1.0010 (1.0000, 1.0020)	4.95E-02^*^	6.35%	216.893	TRUE	1.05E-46	Emilsson [31]
Plasma	OLFM1	Q99784	rs11103667	C	1.0032 (1.0008, 1.0057)	9.71E-03^*^	1.12%	36.376	TRUE	7.29E-09	Emilsson [31]
Plasma	RSPO3	Q9BXY4	rs2489623	C	1.0029 (1.0015, 1.0043)	3.26E-05^**^	3.55%	121.449	TRUE	4.38E-26	Sun [29]
Plasma	SHANK3	Q9BYB0	rs6010042	G	0.9980 (0.9967, 0.9994)	5.07E-03^*^	4.38%	146.679	TRUE	7.48E-32	Emilsson [31]
Plasma	CBLN4	Q9NTU7	rs74447607	T	0.9978 (0.9957, 0.9999)	3.89E-02^*^	1.45%	48.6864	TRUE	1.37E-11	Sun [29]
Plasma	TMEM106B	Q9NUM4	rs10950398	A	0.9980 (0.9966, 0.9993)	3.26E-03^*^	3.57%	118.374	TRUE	6.19E-26	Emilsson [31]
Plasma	EDAR	Q9UNE0	rs6750059	C	0.9988 (0.9978, 0.9997)	1.24E-02^*^	7.49%	80.6158	TRUE	2.51E-18	Suhre [28]
CSF	CPE	P16870	rs11736871	T	1.0147 (1.0009, 1.0287)	3.66E-02^*^	4.51%	39.4497	TRUE	8.54E-10	Yang [23]
CSF	Galectin-3	P17931	rs76426991	G	0.9906 (0.9835, 0.9977)	1.01E-02^*^	17.83%	181.181	TRUE	5.38E-38	Yang [23]
CSF	FUT5	Q11128	rs78114888	G	1.0053 (1.0013, 1.0093)	9.07E-03^*^	22.50%	242.363	TRUE	2.47E-49	Yang [23]

PVE, proportion of variance explained.

^a^All SNPs used were cis-acting.

^b^Odds ratios for increased risk of MS were expressed as per SD increase in plasma protein levels and per 10-fold increase in CSF protein levels.

^*^Statistical significance was considered as suggestive associations (p < 0.05).

^**^Statistical significance was corrected by Bonferroni correction (p < 0.05/888 = 5.63e-05).

### External validations of potential drug targets for EM

**Figure 4:**
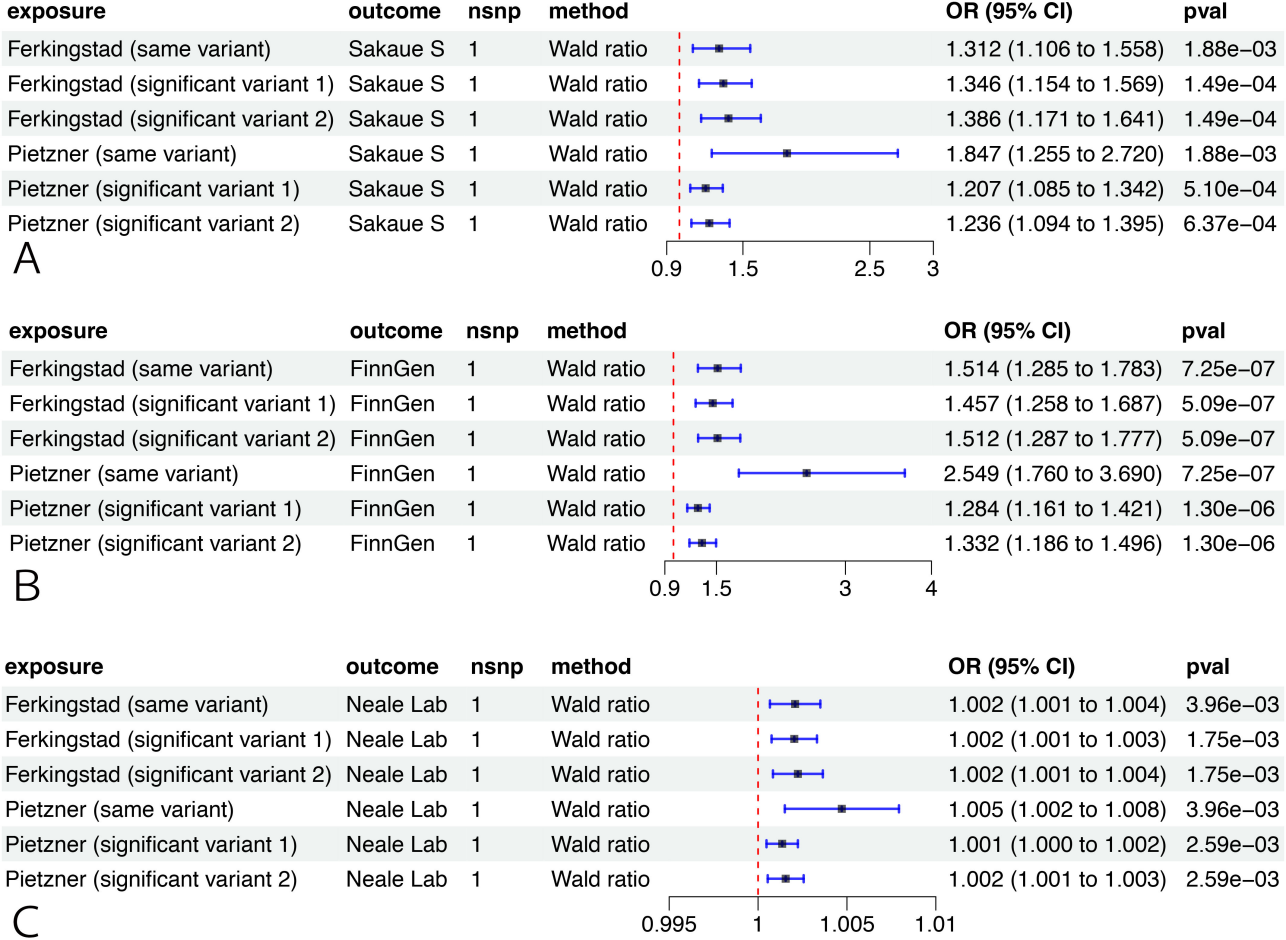
External validation of the causal relationship between RSPO3 and EM. MR analysis on the causal relationship of RSPO3 on EM using data from Sakaue et al. **(A)**, the FinnGen cohort **(B)**, and the UK Biobank **(C)**. OR for increased risk of EM were expressed as per SD increase in plasma protein levels and per 10-fold increase in CSF protein levels. MR, Mendelian randomization; CSF, cerebrospinal fluid; EM, endometriosis; OR, odds ratio; SD, standard deviation.

Applying the same- and significant-variant strategies in different datasets, we replicated our previous findings and confirmed that RSPO3 had a significant association with EM pathogenesis in three cohorts (FinnGen, UK Biobank, and Sakaue et al.) (**Figure 4**).

### Comparisons of target protein levels in plasma and CSF

We found a negligible negative correlation between the CSF and plasma levels of target proteins in MR analysis (Spearman, −0.13; number of proteins (n) = 65). Even after restricting the number of proteins with a varying P-value threshold, we observed a non-significant negative correlation (**Supplementary Figure S1**).

### Associations between drug targets of EM medications and suggestive causal proteins

**Figure 5:**
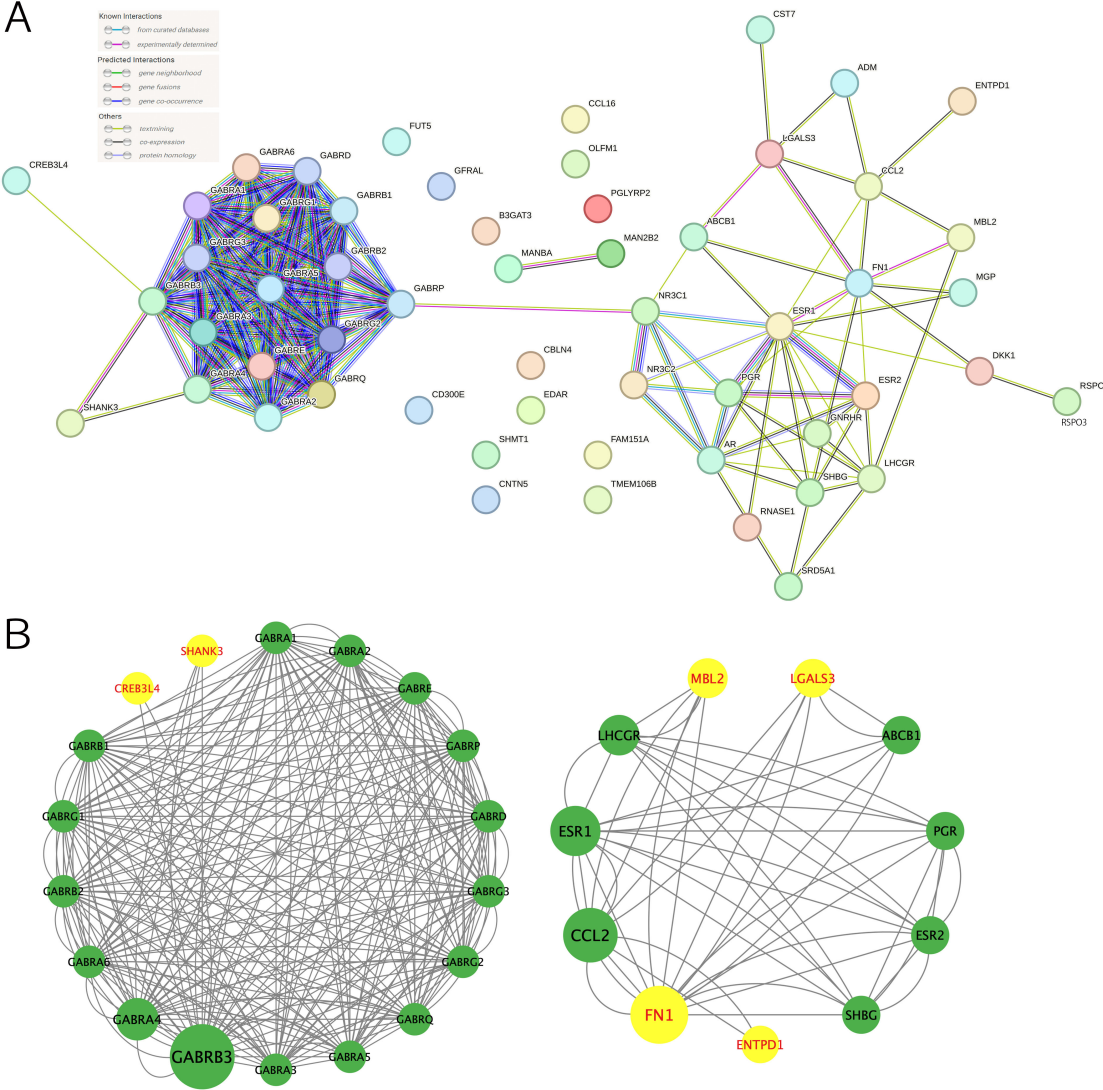
Protein-protein interaction (PPI) networks. **(A)** PPI network of the drug targets of current medications, **(B)** PPI network of drug targets of current medications and suggestive causal proteins (P < 0.05). Yellow represented the suggestive causal proteins.

The 28 drug targets of 23 EM medications are shown in **Supplementary Table S5**. The PPI network displayed interactions between 28 drug targets from 23 EM medications and their interacted proteins (**Figure 5A**). STRING analysis also exhibited interactions between suggestive causal proteins identified from MR analysis (P < 0.05) and drug targets of EM medications. For instance, suggestive causal proteins cyclic AMP-responsive element-binding protein 3-like protein 4 (CREB3L4) and SH3-multiple ankyrin repeat domains protein 3 (SHANK3) proteins could interact with drug targets such as the GABA family proteins; suggestive causal protein LGALS3 could interact with drug targets such as ATP-dependent translocase (ABCB1) and C-C motif chemokine 2 (CCL2); suggestive causal protein ectonucleoside triphosphate diphosphohydrolase 1 (ENTPD1) could interact with drug target like CCL2; suggestive causal protein mannose-binding protein C (MBL2) could interact with drug targets including lutropin-choriogonadotropin hormone receptor (LHCGR) and CCL2; and suggestive causal protein fibronectin (FN1) could interact with drug targets including sex hormone-binding globulin (SHBG), progesterone receptor (PGR), estrogen receptor beta (ESRβ), estrogen receptor 1 (ESR1), ABCB1 and CCL2 (**Figure 5B**). Among them, FN1 and ESR1 had the highest combined score of 0.969 (**Supplementary Table S6**).

### Functional enrichment analyses

The GO enrichment analysis showed that the genes of 29 proteins were mainly enriched in various functions, such as extracellular region, extracellular space, positive regulation of Wnt signaling, planar cell polarity pathway, receptor binding, and sprouting angiogenesis (**Supplementary Figure S2**)

The KEGG analysis also displayed the enrichment of genes of EM-linked proteins MAN2B2 and MANBA in the glycan degradation pathway (**Supplementary Table S7**).

## Discussion

EM is a complex gynecologic pathology involving various genetic, epigenetic, and steroidogenic modulation mechanisms (41). This study was the first to apply a two-sample MR approach using both plasma and brain proteomic datasets to explore protein factors associated with EM. Our MR analysis revealed that plasma-specific RSPO3 had a causal effect on EM. RSPO3 shared the same genetic variant with EM. Additionally, LGALS3, CPE, and FUT5 in CSF were detected as potential targets for EM. PPI analysis showed that FN1 had the highest combined score in the network. Furthermore, several EM-linked proteins were involved in the glycan degradation pathway.

RSPO3 is a secretory protein that plays a crucial role in the Wnt signaling pathway. Zubrzycka et al. (41) found that *WNT4* and *VEZT* genes were most consistently associated with EM pathology. Although RSPO3 was not specifically mentioned in this study, it is worth noting that the Wnt signaling pathway was reportedly dysregulated in EM. Furthermore, Rahmioglu et al. (42) identified RSPO3 as one of the key genes associated with EM susceptibility in a GWAS analysis, suggesting that genetic variations in RSPO3 might contribute to the development of EM. In our study, RSPO3 was the only potential drug target that passed the Bonferroni correction. The PPI results did not show any interactions of RSPO3 with drug targets of current EM medications. GO enrichment showed that RSPO3 had functions in the extracellular region, positive regulation of Wnt signaling, planar cell polarity pathway, receptor binding, and sprouting angiogenesis, which might cause EM, and in addition, played roles in angiogenesis and thrombin formation (43, 44). Therefore, we speculated that RSPO3 might be a promising drug target for EM. Further research is warranted to elucidate the exact role of RSPO3 in EM pathogenesis.

Our PPI results showed that FN1, a suggestive causal protein associated with EM, had interactions with many drug targets of current medications, including SHBG, PGR, ESR2, ESR1, ABCB1, and CCL2. FN1 is a glycoprotein involved in cell adhesion, migration, and tissue remodeling (45). FN1 is implicated in several malignancies, including cervical cancer, gastric cancer, and breast cancer (45–47). FN1 is robustly associated with EM (48). Meta-analysis of GWAS has identified FN1 variants, such as rs1250248, as potential risk factors for EM (49, 50). Sapkota et al. (48) suggested that FN1 might be involved in EM pathogenesis through its role in hormone metabolism. EM is influenced by several hormonal factors, particularly estrogen, and FN1 is shown to interact with ESR1. The PPI results also revealed that FN1 mainly interacted with hormones. Additionally, FN1 regulates cytokine release and several growth factors, including vascular endothelial growth factor A (VEGF-A), which plays a role in endometrial tissue growth and angiogenesis (49). Further investigations are warranted to explore the potential therapeutic implications of targeting FN1 in EM treatment.

KEGG analysis indicated that EM pathology might have a connection with an altered glycan degradation pathway, which was a complex process involving many glycan-degrading enzymes (51). Dysregulation of this pathway can lead to defective autophagy, contributing to the etiology of other diseases (52, 53). The endometrium of EM-affected females expresses higher levels of glycans than that of a healthy individual (54). Furthermore, alterations in glycosylation patterns are observed in advanced-stage EM, demonstrating a potential role of glycan changes in EM pathogenesis (55).

LGALS3 is the only protein showing a suggestive association with EM in both plasma and CSF datasets. LGALS3 is a potential diagnostic and prognostic biomarker of several diseases, including cardiovascular, kidney disease, and cancer (56). LGALS3 is highly expressed and secreted by macrophages, where it regulates alternative macrophage activation (57). Our MR revealed that plasma and CSF LGALS3 had opposite effects on EM (OR = 1.0011; 95% CI: 1.0003–1.0019; P = 0.0101, and OR = 0.9906; 95% CI: 0.9835–0.9977; P = 0.0101, respectively). Therefore, increased LGALS3 plasma level might potentiate the risk of EM, while the upregulation of this protein in CSF might decrease the EM risk. A recent study has found that LGALS3 inhibitor can attenuate allodynia in a mouse model of Alzheimer’s disease (AD) (58), suggesting that sensory neuron-derived LGALS3 may promote allodynia through the microglial secretion of pro-nociceptive mediators. Notably, pain is one of the main symptoms women with EM, and central changes occur in EM-associated pain (59). Therefore, we speculate that LGALS3 may serve as a pain relief target in EM patients.

In the CSF, CPE, and FUT5 also showed significant associations with EM pathology. CPE plays a crucial role in the processing of neuropeptides (60) and is involved in various physiological functions, including embryonic development, signal transduction, and protein processing (60, 61). It also protects neurons against oxidative stress-induced apoptotic cell death (62). Fan et al. demonstrated that neuron-specific CPE knockout could lead to central nervous system dysfunction, including cognitive and motor defects in mice. These results suggest that CPE may be involved in anxiety disorder in EM patients. FUT5 is one of the 13 functionally distinct fucosyltransferase (FUT) genes found in humans (63). FUTs are enzymes responsible for the addition of fucose to glycan structures, and their dysfunction is implicated in many diseases (64, 65) and cancers (66–68). However, the exact function of FUT5 in EM pathology needs to be further explored.

### Limitations

This study has several limitations. First, we examined the disease-causing effects of proteins identified in various studies, and the inconsistent measurements of protein levels across these studies might have introduced biases in the results. However, all circulating proteomic data from GWAS (26, 29, 31, 33) followed aptamer-based analysis. Second, all significantly associated proteins lacked trans-pQTL but had only one cis-acting SNP, which limited the scope of detailed analysis, such as alternative MR algorithms, pleiotropy tests, and heterogeneity assessments. Third, we identified only one target that passed Bonferroni correction (P < 5.63 × 10^−5^), prompting us to perform additional analyses on suggestively associated targets. Fourth, since our analysis focused on the population of European ancestry, it might not be appropriate to generalize the results of this study to other ancestries. Lastly, we observed a wide range of OR across the primary and validation analyses, and the OR was close to 1 in the primary analysis, the OR was close to 1, indicating that there might be limited effects on EM. This phenomenon may be attributed to low protein levels, as most current GWAS data for various diseases are derived from blood samples, which can result in smaller OR values. Despite these, some OR values were larger in the validation cohorts, confirming that RSPO3 was significantly related to EM. Future *in vivo* and *in vitro* studies are still needed to further validate the associations in disease tissues.

## Conclusions

This integrative study suggests that the inherent levels of circulating RSPO3 may be causally associated with EM risks. The protein targets identified in our analysis may have promising applications as potential drug targets for EM. FN1 and glycan degradation pathways may be involved in the development and progression of EM. CSF LGALS3 may serve as a target for pain relief in EM patients. Further studies are warranted to uncover the precise roles of these candidate proteins in EM.

## Data Availability

Publicly available datasets were analyzed in this study. GW summary stats for cis-pQTL can be found in original studies, and that for EM can be retrieved from https://gwas.mrcieu.ac.uk/datasets/. Access to the FinnGen (R9) is available at https://www.finngen.fi/en/access_results.

## Supplementary material

Supplementary Figure 1 Comparison analysis of MR estimates between plasma proteome and brain proteome. **(A)** The study incorporated all 65 shared proteins present in both plasma and brain for conducting correlation analysis. The horizontal and vertical gray lines were indicative of the 95% confidence interval surrounding the MR estimates in the primary analysis. The Spearman correlation coefficient was found to be -0.130 (95% CI: -0.404, 0.173). **(B)** Spearman correlation coefficients were calculated using varying P-value cutoffs to encompass MR estimates. The figures situated to the left of the black data point represented the corresponding counts of shared proteins. MR, Mendelian randomization; 95% CI, 95% confidence interval.

Supplementary Figure 2 GO enrichment analysis for suggestive causal proteins (P < 0.05). GO, Gene Ontology.
