# Supplementary figures and images for: Identification of potential druggable targets for endometriosis through Mendelian randomization analysis
Source: Front Endocrinol (Lausanne). 2025 Jan 22;15:1371498. doi: 10.3389/fendo.2024.1371498 (PMC11794050; doi:10.3389/fendo.2024.1371498)

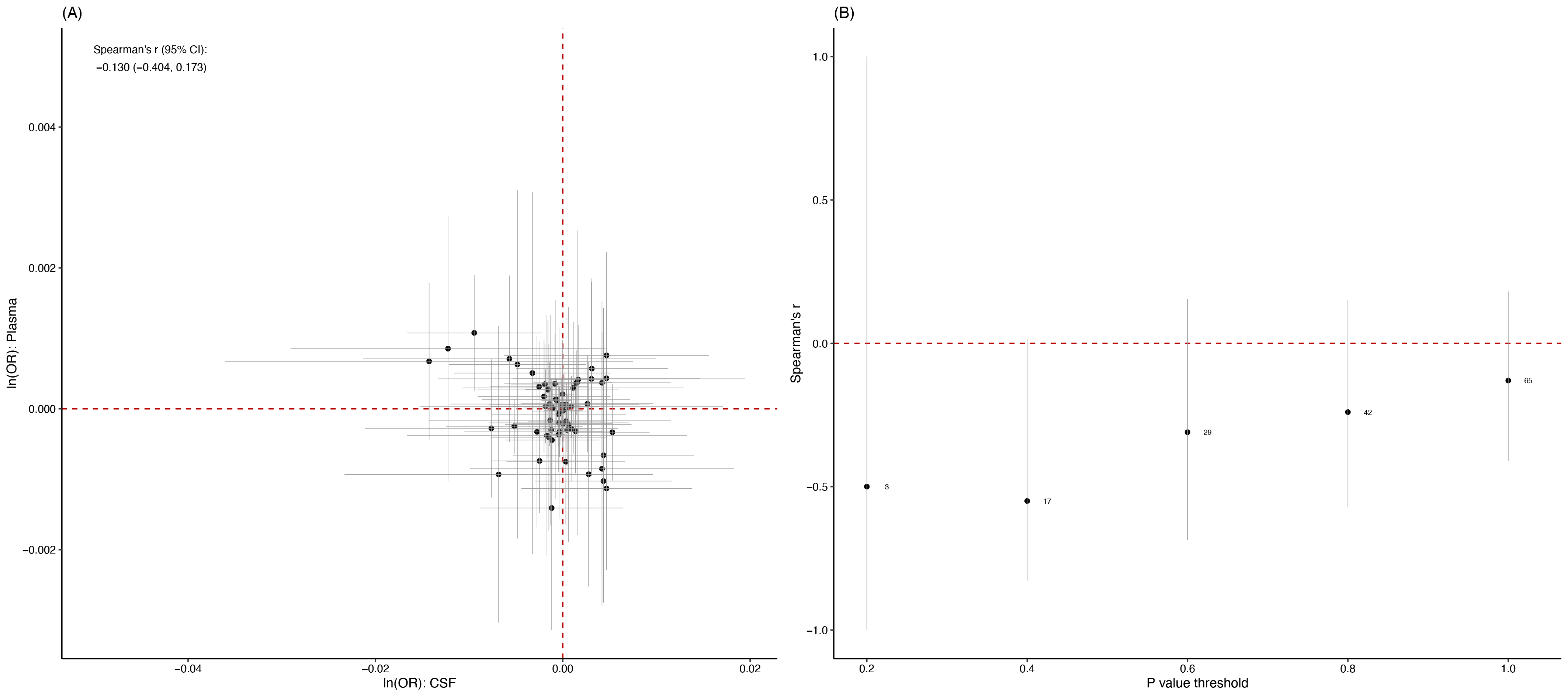

Supplement: Supplementary Figure 1 — Comparison analysis of MR estimates between plasma proteome and brain proteome. (A) The study incorporated all 65 shared proteins present in both plasma and brain for conducting correlation analysis. The horizontal and vertical gray lines were indicative of the 95% confidence interval surrounding the MR estimates in the primary analysis. The Spearman correlation coefficient was found to be -0.130 (95% CI: -0.404, 0.173). (B) Spearman correlation coefficients were calculated using varying P-value cutoffs to encompass MR estimates. The figures situated to the left of the black data point represented the corresponding counts of shared proteins. MR, Mendelian randomization; 95% CI, 95% confidence interval. [file Image1.jpeg]

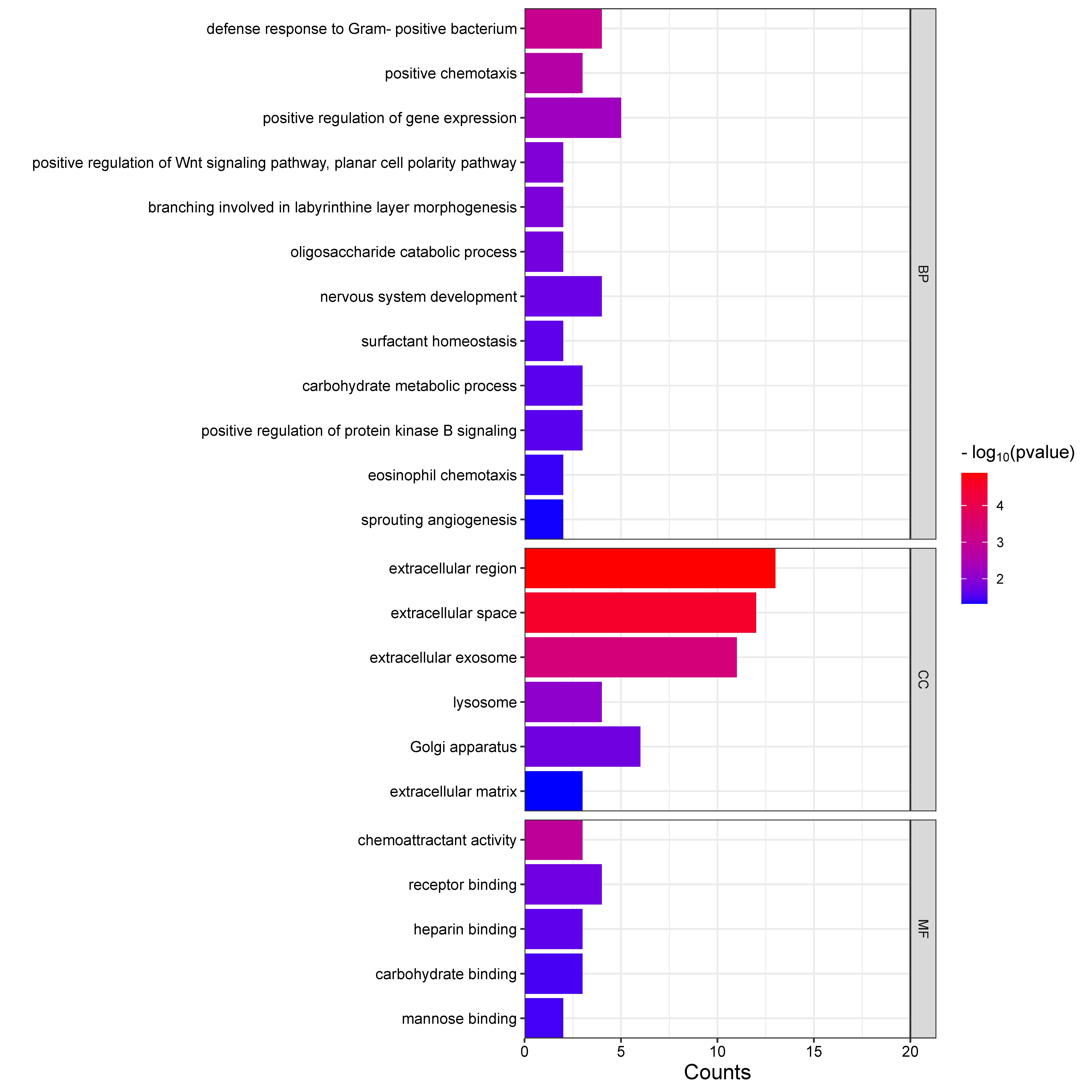

Supplement: Supplementary Figure 2 — GO enrichment analysis for suggestive causal proteins (P < 0.05). GO, Gene Ontology. [file Image2.jpeg]
